# Novel Insights into the Social Functions of the Medial Prefrontal Cortex during Infancy

**DOI:** 10.1523/ENEURO.0458-24.2025

**Published:** 2025-05-21

**Authors:** Tobias Grossmann

**Affiliations:** Department of Psychology, University of Virginia, Charlottesville, Virginia

**Keywords:** brain development, prefrontal cortex, social cognition, social interaction

## Abstract

The medial prefrontal cortex (mPFC) is thought to play a central role in human social perception, cognition, and behavior. In adults, the mPFC is involved in representing and interpreting the mental states in self and others. Developmental research using neuroimaging techniques like functional near-infrared spectroscopy and functional magnetic resonance imaging has begun to extend these findings into infancy. Novel evidence reviewed in this opinion demonstrates that infant mPFC (1) plays a specialized, proactive, and evaluative role in social perception, (2) is involved in connecting with other minds while interacting and when watching other minds interact, and (3) predicts overt social behavior beyond infancy. These findings suggest that, from early in human ontogeny, the mPFC plays a multifaceted role in social perception, cognition, and behavior.

## Significance Statement

This opinion synthesizes recent neuroimaging evidence demonstrating that the medial prefrontal cortex (mPFC) is functionally specialized and actively engaged in social perception, cognition, and behavior starting from infancy. Findings reveal that the infant mPFC proactively processes socially relevant information, predicts emerging social behaviors, and supports both direct and observed social interactions. These novel insights critically add to our understanding of developing brain function and underscore the foundational significance of mPFC for early human social development.

## Introduction

The medial prefrontal cortex (mPFC) plays a central role in human social cognition. Research with human adults has shown that the mPFC is involved in a variety of social cognitive processes, especially when thinking about the psychological attributes of self and others (Amodio and Frith, 2006). Subsequently, a body of developmental social neuroscience research with human infants has demonstrated that the mPFC is active from early in development, supporting social perceptual and cognitive processes in the developing human brain (see Grossmann, 2013, for a review). While previously thought to be functionally inactive in infancy (see Chugani, 2018, for a review arguing that it is not until 1 year of age that infant blood glucose levels increase in medial portions of the prefrontal cortex), studies using functional near-infrared spectroscopy (fNIRS) demonstrate that infants’ mPFC responds sensitively to infant-directed facial and vocal social stimuli from early in the first year (see Grossmann, 2013, for a review).

Following this progress based on the utilization of fNIRS, Deen et al. (2017) obtained face-sensitive responses in the mPFC of 4–6-month-old infants using functional magnetic resonance imaging (fMRI). This further supports the notion of the mPFC playing a specialized role in processing social information from early in human development. This resulted in a conceptual proposal stipulating that the mPFC plays a role in processing faces as more than just a class of visual stimuli but also as signals of social interaction (Powell et al., 2018). Specifically, considering that infants’ mPFC response to faces is strongest when the faces are dynamic and include cues of positive social attention like direct gaze and smiling and when the social interaction is contingent suggests that the mPFC may be involved in evaluating the social relevance of faces and thereby facilitate social engagement early in development (Powell et al., 2018). According to this proposal, mPFC involvement during face processing may contribute to the development of posterior (occipital and temporal) cortical face areas by directing infants’ attention toward the faces of potential social partners (Powell et al., 2018).

## Opinion

The current opinion serves the function to provide an update based on recent research concerning mPFC function in infancy and its role in developing social cognition. This was partly prompted by the recent publication of a fMRI study by Kosakowski et al. (2024), which identified face-selective activity in mPFC of infants, as young as 2 months old. Utilizing fMRI data from a large sample of awake infants aged 2–9 months, the researchers found that the mPFC exhibited significantly greater activation to videos of faces compared with videos of objects, bodies, and scenes. Confirming and extending prior work using fMRI with infants (Deen et al., 2017), face-selective responses in the mPFC were observed across this age range, supporting the view that the mPFC's involvement in processing faces as socially relevant stimuli emerges in infancy (Powell et al., 2018). In fact, there is also evidence to indicate that the mPFC is involved in face processing even before 2 months of age. Using electroencephalography (EEG) methods, newborns have been shown to display face-specific brain responses localized to various cortical brain regions, including the mPFC (Buiatti et al., 2019).

Beyond these important insights demonstrating the early ontogenetic emergence of mPFC involvement in social information processing, there now exists converging evidence attesting to the sophistication of the mPFC involvement in infant social cognition. EEG research with 4- and 9-month-old infants shows that the mPFC contributes to predictive face processing in infancy, showing anticipatory neural activity before the presentation of a face (Mento et al., 2022). In this study, 4- and 9-month-old infants exhibited a category-specific modulation of the contingent negative variation (CNV), an ERP component reflecting anticipatory activity, with a larger CNV elicited by human voices compared with nonhuman sounds when these cues predicted faces. These face-predictive effects were generated in several cortical regions, including the mPFC (Mento et al., 2022). This study further showed the CNV prior to face onset predicted the amplitude of the face-sensitive ERP component (P400; Mento et al., 2022), elicited over posterior brain regions, providing support for the view that mPFC drives specialized brain processes in face-selective regions in line with Powel et al.'s (2018) abovementioned proposal.

Further evidence for the sophistication of mPFC function in infancy comes from a study using a novel dual-brain fNIRS paradigm (Piazza et al., 2020). This study provides evidence that the mPFC plays a critical role in real-life social interactions during infancy by demonstrating significant neural coupling (intersubject correlation) between infants and adults. Specifically, using intersubject correlation analysis 9- to 15-month-old infants’ mPFC responses displayed neural coupling (intersubject correlation) with an adult experimenter's mPFC responses during naturalistic face-to-face interactions, but not when the dyad was engaged in separate tasks (Piazza et al., 2020). Notably, the infants’ mPFC activity preceded the adults’ mPFC activity, particularly during moments of mutual gaze, suggesting that infants’ brains play a leading and proactive role in the dynamics during such social interactions. These findings replicate and extend previous findings regarding mPFC's role in social interaction from screen-based experimental studies with infants (Grossmann, 2013) into real-life social interactions. Moreover, the findings from this study (Piazza et al., 2020) highlight the importance of the mPFC in actively facilitating dynamic and reciprocal social interactions between infants and adults, challenging the traditional view of early social interaction as primarily driven by adult input.

Infants’ mPFC has also been shown to play a role in social evaluation and person perception (Krol and Grossmann, 2020). In this study, 11-month-old infants’ brain responses were measured while they watched videos of four different individuals displaying either smiles or frowns, combined with direct or averted gaze. Following this impression formation phase, infants’ looking preferences for the individuals (now displaying neutral expressions) were assessed using eye-tracking. The findings from this study showed that infants’ mPFC responses discriminated between smiling and frowning individuals only when the faces exhibited direct gaze (Krol and Grossmann, 2020). Furthermore, in this study, infants’ mPFC activity during the impression formation phase predicted subsequent person preferences seen in looking behavior. These findings demonstrate that the mPFC is involved in forming impressions underpinning person perception.

There also exists evidence from longitudinal work showing that mPFC responses during infancy predict later social behavior in toddlerhood (Grossmann and Allison, 2024). This study used fNIRS to show that greater mPFC activity in response to social smiles, but not frowns, at 11 months predicted higher levels of sociability at 18 months. This finding suggests that early variability in mPFC response during positive social interactions is linked to individual differences in overt social behavior. The finding that infant mPFC activity predicts later sociability suggests that early neural responses to social cues may serve as a biomarker for emerging social tendencies rather than directly driving later behavior. A developmental mechanism could involve the mPFC's role in reinforcing positive social experiences, shaping early social motivation and engagement through repeated interactions (Grossmann and Allison, 2024).

Finally, recent work measured brain responses using fNIRS in infants aged 6–13 months as they viewed videos of third-party social interactions and compared the brain responses with when infants viewed two individual actions and inverted social interactions as control conditions (Farris et al., 2022). The results of this study demonstrate that infants exhibited greater activation in the mPFC when observing third-party social interactions compared with both control conditions. In conjunction with the prior work discussed above (Piazza et al., 2020), this suggests that infants’ mPFC plays a role in flexibly supporting both first-person and third-person social interaction processing.

In summary, this suggests that mPFC (1) plays a role in social perception, starting early in human infancy (Buiatti et al., 2019; Kosakowski et al., 2024), (2) serves predictive and social-evaluative functions (Krol and Grossmann, 2020; Mento et al., 2022), (3) is involved in coupling with other brains during first-person social interaction (Piazza et al., 2020) and in processing third-person social interactions (Farris et al., 2022), and (4) longitudinally predicts social behavior beyond infancy (Grossmann and Allison, 2024). This paints a picture of infant mPFC as playing an active and multifaceted role in social perception, cognition, and behavior, earlier and more sophisticated than previously thought. This confirms and critically extends existing conceptual approaches that had hypothesized that mPFC plays a specialized role in social perception (Powell et al., 2018) and in social cognition (Grossmann, 2013) from early in human ontogeny. The novel evidence garnered from developmental social and cognitive neuroscience research with infants (1) challenges late maturational views of prefrontal cortex function and (2) also contests recent proposals, asserting developmental discontinuity between infants’ and adults’ brain functioning (Blumberg and Adolph, 2023; see Liu et al., 2023, for discussion).

Considering this progress, it appears important to flesh out pressing open questions that should be addressed in future work. First, in children and adolescents, the mPFC has been shown to display functional connectivity to other brain regions such as in the superior temporal sulcus and the temporoparietal junction during social tasks (McCormick et al., 2018). It is thus important to examine the functional connectivity from mPFC to other brain regions in infant-friendly, social perceptual, and social cognitive tasks.

Second, while the existing evidence suggests that mPFC function in infants is specialized in processing social cues, it is currently unclear what role more domain-general learning mechanisms might play in accounting for this specialization. For example, a recent study showed that, in infant as young as 3 months of age, mPFC exhibited increased activity in response to structured versus random sequences of (nonsocial) visual stimuli, particularly during the second half of exposure to the structured sequences (Ellis et al., 2021). This learning-related activity in the mPFC is consistent with its role in learning and memory processes in adults, suggesting that the mPFC, in concert with the hippocampus, supports statistical learning of nonsocial stimuli infancy. In this context, it is important to note that in a large meta-analysis of adult fMRI studies (Gilbert et al., 2006), memory processes were associated with more lateral activations of the mPFC, whereas studies involving social cognition (perspective taking and mentalizing) were associated with more medial activations of mPFC. In future research, it will thus be important to assess and contrast statistical learning for social compared with nonsocial stimuli to determine whether social processing recruits specific subsections of the mPFC in infancy.

Third, while existing work suggests that mPFC plays a role in predictive processing (Mento et al., 2022), it remains to be seen whether mPFC modulates activity in posterior (face-selective) brain regions in a top–down manner as seen in adults using dynamic causal modeling from fMRI (Summerfield et al., 2006). While top–down cortical modulation in posterior brain regions has been studied in infants using fNIRS (Emberson et al., 2015), to date, there are no studies including measures from mPFC, which will be a critical extension of existing research.

Fourth, fMRI research with adults indicates that there are anatomically distinct subsections of the mPFC that map onto partly distinct processes involved in social perception, cognition, and behavior (Gilbert et al., 2006; Bzdok et al., 2013; Lieberman et al., 2019). It is thus important to more precisely map social functions in mPFC during infancy using high-resolution techniques, now also available for fNIRS (Collins-Jones et al., 2024), which is more infant-friendly than fMRI in its application as it is less restrictive in terms of noise, movement, etc. while allowing for a testing environment that better approximates infants’ real-life social experiences. Employing a high-resolution mapping approach to mPFC function and systematically comparing between social and nonsocial conditions across age during infancy in future research will enable the examination of functional specialization (domain-specificity) for processing social information.

Fifth, current evidence suggests that mPFC coupling (intersubject correlation) between infants and adults during social interactions (Piazza et al., 2020) reflects a shared neural state, but the precise functional significance of this coupling remains to be fully understood. One possibility is that neural coupling in mPFC plays a critical role in shaping real-time social interactions, actively facilitating shared attention and communication. Alternatively, it may primarily reflect a correlate of shared experience, without exerting a causal influence on interaction dynamics. Future work should explore whether individual differences in mPFC coupling predict specific aspects of social exchanges, such as turn-taking, joint attention, or responsiveness, which could help clarify its mechanistic role. Additionally, experimental designs that manipulate the degree of interactional contingency (e.g., live vs prerecorded or delayed interactions) could provide further insights into whether mPFC coupling drives, rather than merely reflects, social coordination. Given the foundational role of the mPFC in social cognition from early infancy (see Grossmann, 2013, for a review), understanding the functional relevance of neural coupling in this region will be crucial for advancing theories of early social brain development.

## Conclusion

Taken together, the current opinion suggests that the mPFC appears to serve as a key region in the human brain playing a multifaceted role in social perception, cognition, and behavior from early in human ontogeny. This supports the notion that humans are extraordinarily social primates (Tomasello et al., 2012), whose brains have evolved to actively learn, adapt, and benefit from living in interdependent, cooperative, and complex cultural groups.

## Synthesis

Reviewing Editor: Anne Keitel, University of Dundee Decisions are customarily a result of the Reviewing Editor and the peer reviewers coming together and discussing their recommendations until a consensus is reached. When revisions are invited, a fact-based synthesis statement explaining their decision and outlining what is needed to prepare a revision will be listed below. The following reviewer(s) agreed to reveal their identity: Heather Kosakowski, Halie Olsen.

The manuscript has been reviewed by two experts and then discussed with the editor. Both reviewers had some concerns regarding the novelty and thoroughness of the manuscript. Particularly, the description of mPFC as "social hub" needs a clearer definition, more supporting research, and a contrasting with alternative hypotheses (see reviews below). We therefore reached the decision "Revise and Resubmit".

You find the unabridged reviewer comments below. Please respond to each comment in a point-by-point manner.

*****

Reviewer #1

Advances the Field (Required)

The authors are re-stating ideas that have already been stated elsewhere without sufficient justification for the update and without sufficiently engaging in alternative hypotheses.

Comments to the Authors (Required)

The author(s) argue that mPFC is a "hub" for social cognition early in infancy by building on Powell et al., 2018 and incorporating more recent empirical work in the field. Specifically, the authors propose that mPFC:

1) plays a specialized, proactive and evaluative role in social perception

2) is involved in coupling with other minds while interacting and when watching other minds interact

3) predicts overt social behavior beyond infancy

However, there are several open questions that the authors do not address and that leave me confused about the main claims the authors are trying to make. Further, it is not clear that their hypothesis is supported given the cited literature. Finally, the authors do not engage in any possible alternative accounts of the reported literature. The authors need to make significant updates to the manuscript to address these problems.

First, the main claim that mPFC is a social hub in early infancy is not supported by the data presented. The strongest empirical work that the authors have to support their claims are that mPFC activity in older infants is correlated with behavior (social interaction in Piazza et al., 2020, differential looking times in Krol &Grossmann, 2020, and later emerging social measures in Grossmann &Allison 2024). However, there is a lot of developmental change, both behaviorally and anatomically, between early infancy (neonates cited in Buiatti et al., 2019 and 2-month-olds cited in Kosakowski et al., 2024) and later infancy (9-15 months in Piazza et al., 2020; 11-months in Krol &Grossman, 2023; Grossman &Allison, 2024). For example, there are several theories that infants' looking to faces early in infancy can be explained as a salience response (Several papers by Johnson and colleagues). The authors themselves cite Ellis et al, 2021, which is empirical evidence that supports the salience hypothesis over the social hypothesis. Further, there are known anatomical connections between mPFC and subcortical structures (e.g., Roberts et al., 2007; Eblen &Graybiel, 1995; and many others) that are thought to support salience detection in human and non-human primates (e.g., Seeley, 2017). Behaviorally, empirical work shows that young infants will look for similar amounts of time to salient objects and faces (e.g., Kwon et al., 2016). Thus, it is completely possible that face responses in mPFC of young infants are in-fact salience responses and not at all social in nature.

The future directions section is also quite confusing. What do the authors mean when they say the "mPFC has been shown to operate in concert with other brain regions such as the superior temporal sulcus and the temporo-parietal junction during social tasks" and how is this relevant to the claims the authors are trying to make? Why does "rest-state measurement of functional brain network connectivity" matter at all? Especially in infancy?

The authors state that "mPFC modules activity in posterior brain regions in a top-down manner .. in adults." What does this statement mean and how is it relevant to the hypothesis that mPFC is a social hub in infancy?

Finally, the authors themselves point out that, "fMRI research with adults indicates that there are anatomically distinct subsections of the medial prefrontal cortex that map onto partly distinct processes involved in social perception, cognition and behavior..." Given the authors believe this, how can they make the claim that in infancy mPFC is a social hub? Do they think that mPFC starts as a so-called "social hub" and then divides into different anatomically distinct functions. Given they suggest that future research should use high resolution fNIRS, it doesn't seem like that is the claim they are trying to make. Rather, it seems like the authors believe that mPFC may already form anatomically distinct regions that support different functions, even early in infancy. If so, how is that idea consistent with the main claim that mPFC is a hub that supports social function in infancy?

Smaller points that should be addressed:

1) The authors never define what a hub is. The term "hub" has come to mean different things to different research groups where some focus on anatomical connections and others focus on functional integration. The authors use the work "hub" without defining it and without making a clear claim.

2) Please provide a reference for the claim that mPFC was:

"... previously thought to be underdeveloped in infancy..." and explain what you mean by it.

3) When citing Grossman (2013, 2015, and 2017), please make it clear that they are opinions/reviews, not empirical papers.

4) Deen et al., 2017 did not observe "face-selective" responses. "selective" generally means that the responses to one condition is greater than the response to every other condition. Deen and colleagues report a face response that is greater than the response to scenes but they never report if the face response is also greater than the responses to bodies and objects. Further, the authors themselves do not claim to find "face-selective" responses in mPFC. (See supplementary Figure 5 from Deen et al., 2017)

Citations:

Eblen, F., &Graybiel, A. M. (1995). Highly restricted origin of prefrontal cortical inputs to striosomes in the macaque monkey. Journal of Neuroscience, 15(9), 5999-6013.

Kwon, M. K., Setoodehnia, M., Baek, J., Luck, S. J., &Oakes, L. M. (2016). The development of visual search in infancy: Attention to faces versus salience. Developmental psychology, 52(4), 537.

Seeley, W. W. (2019). The salience network: a neural system for perceiving and responding to homeostatic demands. Journal of Neuroscience, 39(50), 9878-9882.

Roberts, A. C., Tomic, D. L., Parkinson, C. H., Roeling, T. A., Cutter, D. J., Robbins, T. W., &Everitt, B. J. (2007). Forebrain connectivity of the prefrontal cortex in the marmoset monkey (Callithrix jacchus): An anterograde and retrograde tract‐tracing study. Journal of Comparative Neurology, 502(1), 86-112.

*****

Reviewer #2

Advances the Field (Required)

The present manuscript provides a timely review of recent neuroimaging evidence (from fNIRS, EEG, and fMRI) demonstrating the role of medial prefrontal cortex (mPFC) in infant social cognition. MPFC has long been known to be involved in adult social cognition, and over the past ~15-20 years more and more evidence has shown that mPFC is also involved in social perception and cognition during infancy. This opinion paper particularly focuses on work from the last ~5-6 years that together provide a more detailed understanding of the role of mPFC in infant social cognition, and point to more sophistication of mPFC function in infancy than previously assumed. While this opinion piece does not put forth a completely novel idea, I do think that it clearly summarizes recent relevant work and synthesizes current understanding of the role of mPFC in social cognition in infancy.

Comments to the Authors (Required)

The authors provide a timely opinion piece focused on recent neuroimaging evidence (from fNIRS, EEG, and fMRI) that points to a sophisticated role of medial prefrontal cortex (mPFC) in infant social cognition. They focus on three lines of evidence - (1) that infant mPFC plays a specialized role in social perception, (2) that infant mPFC is involved in "coupling with other minds" during interaction, and (3) that infant mPFC predicts social behavior beyond infancy - to make the claim that mPFC is a "hub" for social cognition early in development.

Overall, I found the paper clear, compelling, and timely. I have minor comments that the authors may wish to consider in the revision.

* I think this line would benefit from references: "While previously though to be underdeveloped... such as faces and voices" (lines 33-34) [unless this is is referring to references in the previous sentence?]

* The Piazza et al (2020) study looked at infant-adult neural coupling, but the adult was not the child's caregiver - I believe they had the same adult experimenter for all of the dyads. This should be fixed in line 81 of the paper which says that "mPFC responses were coupled with their caregiver's mPFC responses"

* I find the mPFC neural coupling evidence both relevant and interesting, and perhaps also worth mentioning in the "future directions" section because I think there are many unanswered questions. Does neural coupling mean that mPFC is playing a "critical role" in these interactions, or merely reflecting something about the shared experience? Do differences in neural coupling in mPFC predict features of the interaction? While I certainly think it means something that this similarity in processing happens in mPFC, the actual interpretation of what neural coupling means from a mechanistic standpoint is not completely clear (not a criticism of this paper, but just a general open question).

* With such a short paper, it is not possible to fully flesh out each line of evidence, but I felt that the third line of evidence (infant mPFC predicts social behavior beyond infancy) was a bit underdeveloped in terms of the overall argument. Is there a hypothesized developmental mechanism here, or is the claim about mPFC activity being a biomarker for "sociability"? Not critical to address if it doesn't fit into this short paper, but was left wondering about this piece of evidence in the broader scope of this opinion piece.

* I wonder whether the authors have room to slightly expand on this claim: "also contests recent proposals, asserting developmental discontinuity between infants' and adults' brain functioning" (line 132) - fine if not, but I agree that this evidence makes a compelling case for developmental continuity of mPFC.

* I think that the open questions section is a strength of this paper, identifying clear next steps for research in this domain.

## References

[B1] Amodio DM, Frith CD (2006) Meeting of minds: the medial frontal cortex and social cognition. Nat Rev Neurosci 7:268–277. 10.1038/nrn1884 16552413

[B2] Blumberg MS, Adolph KE (2023) Infant action and cognition: what's at stake? Trends Cogn Sci 27:696–698. 10.1016/j.tics.2023.05.008 37321923 PMC10528200

[B3] Buiatti M, Di Giorgio E, Piazza M, Polloni C, Menna G, Taddei F, Baldo E, Vallortigara G (2019) Cortical route for facelike pattern processing in human newborns. Proc Natl Acad Sci U S A 116:4625–4630. 10.1073/pnas.1812419116 30755519 PMC6410830

[B4] Bzdok D, Langner R, Schilbach L, Engemann DA, Laird AR, Fox PT, Eickhoff SB (2013) Segregation of the human medial prefrontal cortex in social cognition. Front Hum Neurosci 7:232. 10.3389/fnhum.2013.00232 23755001 PMC3665907

[B5] Chugani HT (2018) Imaging brain metabolism in the newborn. J Child Neurol 33:851–860. 10.1177/0883073818792308 30112963

[B6] Collins-Jones LH, et al. (2024) Whole-head high-density diffuse optical tomography to map infant audio-visual responses to social and non-social stimuli. Imaging Neurosci 2:1–19. 10.1162/imag_a_00244 PMC12272203 40800512

[B7] Deen B, Richardson H, Dilks DD, Takahashi A, Keil B, Wald LL, Kanwisher N, Saxe R (2017) Organization of high-level visual cortex in human infants. Nat Commun 8:13995. 10.1038/ncomms13995 28072399 PMC5234071

[B8] Ellis CT, Skalaban LJ, Yates TS, Bejjanki VR, Córdova NI, Turk-Browne NB (2021) Evidence of hippocampal learning in human infants. Curr Biol 31:3358–3364.e4. 10.1016/j.cub.2021.04.072 34022155

[B9] Emberson LL, Richards JE, Aslin RN (2015) Top-down modulation in the infant brain: learning-induced expectations rapidly affect the sensory cortex at 6 months. Proc Natl Acad Sci U S A 112:9585–9590. 10.1073/pnas.1510343112 26195772 PMC4534272

[B10] Farris K, Kelsey CM, Krol KM, Thiele M, Hepach R, Haun DB, Grossmann T (2022) Processing third-party social interactions in the human infant brain. Infant Behav Dev 68:101727. 10.1016/j.infbeh.2022.101727 35667276

[B11] Gilbert SJ, Spengler S, Simons JS, Steele JD, Lawrie SM, Frith CD, Burgess PW (2006) Functional specialization within rostral prefrontal cortex (area 10): a meta-analysis. J Cogn Neurosci 18:932–948. 10.1162/jocn.2006.18.6.932 16839301

[B12] Grossmann T (2013) The role of medial prefrontal cortex in early social cognition. Front Hum Neurosci 7:340. 10.3389/fnhum.2013.00340 23847509 PMC3701864

[B13] Grossmann T, Allison O (2024) Dorso-medial prefrontal cortex responses to social smiles predict sociability in early human development. Imaging Neurosci 2:1–8. 10.1162/imag_a_00129 PMC12247620 40800354

[B14] Kosakowski HL, Cohen MA, Herrera L, Nichoson I, Kanwisher N, Saxe R (2024) Cortical face-selective responses emerge early in human infancy. eNeuro 11:ENEURO.0117-0124.2024. 10.1523/ENEURO.0117-24.2024 38871455 PMC11258539

[B15] Krol KM, Grossmann T (2020) Impression formation in the human infant brain. Cereb Cortex Commun 1:tgaa070. 10.1093/texcom/tgaa070 33134930 PMC7592636

[B16] Lieberman MD, Straccia MA, Meyer ML, Du M, Tan KM (2019) Social, self, (situational), and affective processes in medial prefrontal cortex (MPFC): causal, multivariate, and reverse inference evidence. Neurosci Biobehav Rev 99:311–328. 10.1016/j.neubiorev.2018.12.021 30610911

[B17] Liu S, Raz G, Kamps F, Grossmann T, Saxe R (2023) No evidence for discontinuity between infants and adults. Trends Cogn Sci 27:694–695. 10.1016/j.tics.2023.04.003 37321922 PMC10524850

[B18] McCormick EM, van Hoorn J, Cohen JR, Telzer EH (2018) Functional connectivity in the social brain across childhood and adolescence. Soc Cogn Affect Neurosci 13:819–830. 10.1093/scan/nsy064 30085317 PMC6123525

[B19] Mento G, Duma GM, Valenza E, Farroni T (2022) Face specific neural anticipatory activity in infants 4 and 9 months old. Sci Rep 12:12938. 10.1038/s41598-022-17273-1 35902656 PMC9334392

[B20] Piazza EA, Hasenfratz L, Hasson U, Lew-Williams C (2020) Infant and adult brains are coupled to the dynamics of natural communication. Psychol Sci 31:6–17. 10.1177/0956797619878698 31845827 PMC6966249

[B21] Powell LJ, Kosakowski HL, Saxe R (2018) Social origins of cortical face areas. Trends Cogn Sci 22:752–763. 10.1016/j.tics.2018.06.009 30041864 PMC6098735

[B22] Summerfield C, Egner T, Greene M, Koechlin E, Mangels J, Hirsch J (2006) Predictive codes for forthcoming perception in the frontal cortex. Science 314:1311–1314. 10.1126/science.1132028 17124325

[B23] Tomasello M, Melis AP, Tennie C, Wyman E, Herrmann E (2012) Two key steps in the evolution of human cooperation: the interdependence hypothesis. Curr Anthropol 53:673–692. 10.1086/668207

